# Three novel trehalase genes from *Harmonia axyridis* (Coleoptera: Coccinellidae): cloning and regulation in response to rapid cold and re-warming

**DOI:** 10.1007/s13205-019-1839-9

**Published:** 2019-08-06

**Authors:** Zuo-Kun Shi, Shi-Gui Wang, Ting Zhang, Yu Cao, Yan Li, Can Li

**Affiliations:** 1 Guizhou Provincial Key Laboratory for Rare Animal and Economic Insect of the Mountainous Region, Department of Biology and Engineering of Environment, Guiyang University, Guiyang, 550005 People’s Republic of China; 2 College of Life and Environmental Sciences, Hangzhou Normal University, Hangzhou, 310036 Zhejiang People’s Republic of China

**Keywords:** *Harmonia axyridis*, Membrane-bound trehalase, Rapid cold hardening, Soluble trehalase, Trehalose

## Abstract

Trehalose is the main blood sugar in insects. To study the function of trehalase during exposure to low temperatures, three other novel cDNAs of trehalase were cloned from *Harmonia axyridis* by transcriptome sequencing and rapid amplification of cDNA ends. One of the cloned cDNAs encoded a soluble trehalase, the second trehalase cDNA encoded a transmembrane-like domain, and the third cDNA encoded a membrane-bound protein. Therefore, these cDNAs were, respectively, named *HaTreh1*-*5*, *HaTreh2*-*like*, and *HaTreh2*. *HaTreh1*-*5*, *HaTreh2*-*like*, and *HaTreh2* cDNAs encoded proteins containing 586, 553, and 633 amino acids with predicted masses of approximately 69.47, 63.46, and 73.66 kDa, and pIs of 9.20, 5.52, and 6.31, respectively. All three novel trehalases contained signal motifs “PGGINKESYYLDSY”, “QWDYPNAWPP”, and a highly conserved glycine-rich (GGGGEY) region. The expression levels of *HaTreh1*-*5* and *HaTreh2* mRNAs were high during adult stages, whereas *HaTreh2*-*like* was expressed in low amounts in the fourth larval stage. The results showed that the activity of membrane-bound trehalases decreased from 25 to 10 °C and from 5 to − 5 °C during cooling. The results also revealed a decreasing trend in expression of the three *HaTreh* mRNAs during the cooling treatment, and an initial decrease followed by an increase during the process of re-warming.

**Electronic supplementary material:**

The online version of this article (10.1007/s13205-019-1839-9) contains supplementary material, which is available to authorized users.

## Introduction

The disaccharide trehalose consists of two glucose units linked through an α-glycosidic bond, which can synthesize in insect fat body by trehalose-6-phosphate synthase (TPS) and trehalose phosphate phosphatase (TPP) pathway (Caner et al. 2013; Tang et al. 2010; van Wyk et al. 2017; Xu et al. 2009; Yang et al. 2017; Tang et al. 2018). To our knowledge, trehalose is the main sugar in the hemolymph of insects, and exists in all kinds of organisms, except mammals. Its function is very important in insects and fungi (Elbein et al. 2003; Frison et al. 2007; Silva et al. 2010; Wingler 2002; Xu et al. 2009; Tang et al. 2018). Trehalose can be stored as energy and carbohydrates, and it is an agent that protects proteins and cell membranes from various environmental stress conditions, including desiccation, heat, freezing, and oxidation (Caner et al. 2013; Crowe et al. 1984; Eleutherio et al. 1993; Koštál et al. 2014). However, the detailed mechanisms of how trehalose protects insects from cold are still poorly understood, and little information about its effects under rapid cold or re-warming is present. To utilize blood trehalose, insect tissues have trehalases (alpha-glucoside-1-glucohydrolase, EC 3.2.1.28) that catalyze the hydrolysis of one mole of trehalose to two moles of glucose (Silva et al. 2004; Thompson 2003; Shukla et al. 2019; Adhav et al. 2019). Trehalases are thought to be located on the cell membrane or within the cell cytoplasm (Valaitis and Bowers 1993; Yaginuma et al. 1996; Nardelli et al. 2019).

The first study on soluble trehalase in insects involved the purification and assessment of some properties of this enzyme, including genetic and biochemical aspects, in *Drosophila melanogaster*. Only one molecular form of trehalase (E.C. 3.2.1.28) was detectable in adult *D. melanogaster* by polyacrylamide gel electrophoresis and isoelectric focusing (Bargiello and Grossfield 1980; Huber and Lefebvre 1971; Oliver et al. 1978). The first insect trehalase cDNA, a soluble trehalase, was cloned and reported in 1992 from *Tenebrio molitor* (Takiguchi et al. 1992). Although insects were divided into two types of trehalases, soluble (named Treh1, Tre1 or TreS) and membrane-bound (named Treh2, Tre2 or TreM) depending on whether they have potential putative transmembrane helices or domains, while *Treh2* gene was not reported until 2005 (Mitsumasu et al. 2005). Treh2 possesses a transmembrane domain and is usually larger, whereas Treh1 lacks a transmembrane domain (Shukla et al. 2015). Treh1 is a cytosolic enzyme that hydrolyses endogenous trehalose, whereas Treh2 is an extracellular enzyme (Ma et al. 2015). Treh2 is believed to face the blood side, degrading the extracellular trehalose (Mitsumasu et al. 2005). The glucose so generated then enters into the cell and is utilized for physiological activities (Shukla et al. 2015). Moreover, most of the insect species, such as *Spodoptera exigua* (Chen et al. 2010; Tang et al. 2008), *Apis mellifera* (Lee et al. 2007; Mori et al. 2009), *Bombyx mori* (Kamei et al. 2011; Mitsumasu et al. 2005), *Laodelphax striatellus* (Zhang et al. 2012), *Omphisa fuscidentalis* (Tatun et al. 2008a, b), *D. melanogaster* (Shukla et al. 2016), *Chironomus ramosus* (Shukla et al. 2019), and others (Bansal et al. 2013), have genes for one soluble and one membrane-bound trehalase or only one of the trehalases. Two *Treh1* and one *Treh2* genes have been cloned from *Nilaparvata lugens* (Gu et al. 2009; Zhao et al. 2016), four *Treh1* have been cloned from *Harmonia axyridis* (Shi et al. 2016; Tang et al. 2014), four *Treh1* and one *Treh2* have been cloned from *Tribolium castaneum* (Tang et al. 2016). However, not only two *Treh1* and one *Treh2*, but also one *Treh2*-*like* have been cloned from *Locusta migratoria* (Liu et al. 2016), it is the first-reported insect to have a Treh2-like protein.

These trehalase genes have been cloned and studied in almost all the pests because of which they could be employed as potential target genes for insect control (Liebl et al. 2010; Zhao et al. 2016). *Treh1* and *Treh2* are known to regulate wing development, flight metabolism, and chitin biosynthesis, and they also play distinct roles in chitin biosynthesis and other physiological activities (Chen et al. 2010; Shukla et al. 2015). The beetle, *Harmonia axyridis* (Pallas), is an important natural enemy of pests, such as aphids, whiteflies, psyllids, mites, and the eggs and larvae of some lepidopterans and coleopterans (Luciana et al. 2015; Zappalà et al. 2013); it is the focus of pest control strategies in agriculture and forestry in Asia, including China (Tang et al. 2014), but is notorious for its invasiveness of the ecological niche of other ladybird beetles in Europe (Adriaens et al. 2008; Brown et al. 2008a, b). Cold hardiness and diapause are essential components of winter survival for most insects in temperate zones, but in many cases the relationship between these two are not clear (Heydari and Izadi 2014). The cold-hardiness of *H. axyridis* represents an important characteristic and has been widely studied with respect to biological control applications and aspects of invasiveness (Bazzocchi et al. 2004; Berkvens et al. 2010; Pervez and Omkar 2006; van Lenteren et al. 2008). In addition, the genes controlling trehalase were identified as important to low-temperature stress resistance (Shi et al. 2016; Wu et al. 2016). Four soluble trehalase genes (*HaTreh1*-*1* to *HaTreh1*-*4*) have been cloned (Shi et al. 2016; Tang et al. 2014). In the present study, a new soluble trehalase (*HaTreh1*-*5*), one membrane-bound (*HaTreh2*), and more importantly, one like membrane-bound trehalase (*HaTreh2*-*like*) cDNAs from *H. axyridis* were identified and cloned. The results obtained in this study can help understand the response of the trehalase gene to cold stress: (1) withstanding loss of activity at low temperature, (2) protecting cell function and thus surviving a sustained low-temperature exposure, or (3) repairing damage upon re-warming from a chill exposure.

## Materials and methods

### Insect cultures

*Harmonia axyridis* culture was established in our laboratory using insects collected from the Lab of Natural Enemy Research, Beijing Academy of Agriculture and Forestry Science. The experimental population was maintained in the laboratory at 25 ± 1 °C for over 3 years and was fed *Aphis medicaginis*. The developmental stages were synchronized at each molt by collecting new larvae, pupae, or adult by giving fresh *A. medicaginis*, which were feeding on broad bean seedlings. The light period started at 06:00 h and ended at 22:00 h (L16:D8 photoperiod) (Shi et al. 2016).

### Cold and re-warming

Heating and cooling rates really matter for insect survival (Terblanche et al. 2011). The expression of the genes was tested at six temperatures: 25 °C, 15 °C, 10 °C, 5 °C, 0 °C, and − 5 °C. The choice of temperature refers to the study by Wang (Wang et al. 2017). To mimic the cooling conditions under natural conditions, we set a gradient of 5 °C between − 5 and 15 °C. Moreover, a previous study has shown that 5 °C may be the temperature signal of cold stress of *H. axyridis* (Shi et al. 2016). *H. axyridis* individuals were placed in one of the following environments with rapidly changing temperatures: (i) from 25 to − 5 °C, or (ii) from − 5 to 25 °C. The treatment (i) consisted of the following steps: one hundred and twenty individuals were placed in plastic fruit fly tubes sealed with sponge (ten individuals per tube) and were then maintained at 25 °C; after 2 h exposure to 25 °C, the tubes were cooled rapidly to 15 °C, and further cooled to 10 °C after 2 h exposure to 15 °C. Finally, the tubes were cooled to − 5 °C; the treatment (ii) involved a similar procedure, but the starting temperature was set at − 5 °C. The above treatments were repeated three times (Shi et al. 2016). The membrane-bound trehalase activities and gene expression levels were measured at each temperature.

### RNA extraction, cDNA synthesis, and rapid amplification of cDNA ends (RACE)

Total RNA was extracted from adult individuals (with elytra removed) using the TRIzol (Invitrogen, USA) method. The first-strand cDNA synthesis was carried out according to the manual of PrimeScript^®^ RT reagent Kit using gDNA Eraser (TaKaRa). The samples of 1 μg total RNA were reverse-transcribed at 42 °C for 1 h in a 10-μl reaction mixture containing reaction buffer, 10 mM DTT, 0.5 mM dNTPs, 0.5 μg oligo-dT18, and reverse transcriptase from avian myeloblastosis virus (TaKaRa). First-strand cDNA (1 μl) was used as a template for the polymerase chain reaction (PCR).

Partial sequences of the novel soluble and two membrane-bound trehalase genes were obtained by transcriptome sequencing of *H. axyridis*. Using 5′- and 3′-RACE, 5′-ready-cDNA and 3′-ready-cDNA were synthesized according to the manufacturer’s protocol (SMART™ kit, TaKaRa). Specific primers, HaTreh2-like-5RA and HaTreh2-like-5RB for 5′-RACE and HaTreh1-5-3FA and HaTreh1-5-3FB, for 3′-RACE (Table 1), were synthesized based on the cDNA sequence obtained by transcriptome sequencing. 5′-RACE was performed using 2.5 μl of 5′-ready-cDNA and Universal Primer Mix (UPM, TaKaRa) along with HaTreh1-5-5RA and HaTreh2-like-5RA, there after nested PCR was carried out with Nested Universal Primer A (NUP, TaKaRa) along with HaTreh1-5-5RB and HaTreh2-like-5RB. 3′-RACE was performed using 2.5 μl of 3′-ready-cDNA and UPM along with HaTreh1-5-3FA and HaTreh2-like-3FA, followed by amplification using NUP along with HaTreh1-5-3FB and HaTreh2-like-3FB. The PCR conditions used were as follows: 10 min at 94 °C, 30 cycles of 30 s at 94 °C, 30 s at 60 °C, and 120 s at 72 °C, followed by 10 min at 72 °C.

**Table 1:** The primers used in this study

PCR fragments	Primer names	Nucleotide sequences (5′–3′)
RACE	HaTreh1-5-3FA	CCTCCACTCCTCCTAATG
	HaTreh1-5-3FB	CCACACCAAGACCAGAGT
	HaTreh2-like-5RA	TCACATGCCGCTTTGATCTCCGAGT
	HaTreh2-like-5RB	GACCCTCTGAACTGTTACCATAA
QRT-PCR	HaTreh1-5QF	TGATGATGAGGTACGACGAGAA
	HaTreh1-5QR	GTAGCAAGGACCTAACAAACTGC
	HaTreh2-likeQF	TTCCAGGTGGGAGATTCAGG
	HaTreh2-likeQR	GGGATCAATGTAGGAGGCTGTG
	HaTreh2QF	CAATCAGGGTGCTGTAATGTCG
	HaTreh2QR	CGTAGTTGGCTCATTCGTTTCC
Reference gene	Ha-rp49-QF	GCGATCGCTATGGAAAACTC
	Ha-rp49-QR	TACGATTTTGCATCAACAGT

F and R represent forward and reverse primers, respectively

*Ha Harmonia axyridis*, *Treh* trehalase

The products were subjected to agarose gel electrophoresis. The desired DNA fragments were excised from the agarose gel and purified using a DNA gel extraction kit (OMEGA, USA). Purified DNA was ligated into pMD18-T vector (TaKaRa, Japan) and sequenced by dideoxynucleotide method.

### Analysis of the *HaTreh* cDNA and protein sequences

*HaTreh1*-*5* and *HaTreh2* cDNA sequences were compared with other soluble and membrane-bound trehalase sequences present in GenBank using the BLAST-N and BLAST-X tools available on the National Center for Biotechnology Information (NCBI) website. Multiple sequence alignment of insect trehalases was performed using the tool available at the multiple sequence alignment website (http://bioinfo.genotoul.fr/multalin/multalin.html) and using Vector NTI Suite 9. The neighbor-joining method was used to construct a phylogenetic tree based on the amino acid sequences of known Treh proteins using MEGA 6.0 software. Bootstrap analysis was carried out and the robustness of each cluster was verified using 1000 replicates. HaTrehs protein sequences and other analysis criteria used in this study, including MW, pI, and topology were deduced from the corresponding cDNA sequences using the translation tool on the ExPASy proteome prediction tools website (http://expasy.org/tools/dna.html). The analysis of HaTreh signal peptide was performed using “SignalP 4.1 server” available at http://www.cbs.dtu.dk/services/SignalP/.

### Expression level of *HaTrehs* during developmental stages, cooling and re-warming by quantitative real-time polymerase chain reaction (qRT-PCR)

Total RNA was isolated from *H. axyridis* whole body (with elytra removed) on each day of its life cycle, including the fourth instar larvae, pupal, and adult (removal of elytra) stages, as well as total RNA was isolated from the entire body of *H. axyridis* (with elytra removed) during cooling and re-warming treatments. One microgram of total RNA was used as a template to synthesize first-strand cDNA using a PrimeScript RT with gDNA Eraser kit (TaKaRa, Japan). The expression of *HaTreh1*-*5*, *HaTreh2*-*like*, and *HaTreh2* was estimated by qRT-PCR (Suann et al. 2015) using a Bio-Rad CFX96™ system (Bio-Rad, USA) and SsFast™ EvaGreen Supermix (Bio-Rad, USA). Primers were designed to determine the expression of the genes, *HaTreh1*-*5*, *HaTreh2*-*like,* and *HaTreh2* (Table 1). These primers were designed for their own unique regions found in the alignment using Vector NTI Suite 7. Each reaction was performed in a final volume of 20 µl, containing 1 µl of the cDNA sample (or standard), 1 µl (10 µM) of each primer, 7 µl RNAase- and DNAase-free water, and 10 µl of SsoFast™ EvaGreen Supermix. The cycling conditions consisted of 3 min of initial denaturation at 95 °C, 40 cycles of denaturation at 95 °C for 5 s, annealing at 55 °C–62.5 °C for 20 s, and finally a melt curve was made at 65–95 °C, as per the manufacturer’s instructions.

The expression patterns of *HaTreh1*-*5* and *HaTreh2s* mRNAs on each day of the life cycle of *H. axyridis*, from the fourth instar larva through pupa to 3-day-old adult, were determined by real-time quantitative PCR. Also the changes of *HaTrehs* expression level during cooling and re-warming were determined by qRT-PCR. The expression of *H. axyridis* rp49 (ribosomal protein 49 gene) on each day was used to standardize the relative expression levels of these three *Treh* genes (Shi et al. 2016).

### Activity assay for membrane-bound trehalase

To determine the activity of trehalase, the extraction solution, containing membrane-bound trehalase, was reacted, in a pH 6.0 buffer with excess substrates, by incubating at 37 °C (Tatun et al. 2008a, b). Specifically, the whole body of three to five adults (with elytra removed), including male and female individuals, were homogenized in a 1.5-ml tube at 0 °C (TGrinder OSE-Y20 homogenizer, TIANGEN, China) after adding 200 μl of 20 mM phosphate-buffered saline (PBS, pH 6.0), followed by sonication for 30 s (VCX 130 PB, Sonics, USA). The homogenates were centrifuged at 1000×*g* at 4 °C for 10 min after adding 800 μl PBS; the cuticle debris was removed and centrifuged at 105,000×*g* for 60 min at 4 °C (CP100MX, Hitachi, Japan). The supernatant was directly removed and the precipitate fraction was washed twice with PBS, and then suspended in 200 μl PBS for the measurement of membrane-bound trehalase. The amount of protein in each sample was determined prior to the trehalase assay by a protein–dye binding method (Bio-Rad, USA) using bovine serum albumin as standard. The reaction mixture (250 μl) for the membrane-bound trehalase activity assay, consisted of 62.5 μl 40 mM trehalose (Sigma, USA) in 20 mM PBS (pH 6.0), 50 μl membrane-bound trehalase extracts, and 137.5 μl PBS. The mixture was incubated at 37 °C for 30 min, and the reaction was stopped by heating the reaction mixture in boiling water for 5 min. Coagulated protein was removed by centrifugation at 12,000×*g* at 4 °C for 10 min, and an aliquot of the resulting supernatant was used to measure the amount of glucose using a Glucose Assay Kit (GAGO20-1KT, Sigma, USA), following the manufacturer’s instructions. First, glucose was converted into glucose acid and hydrogen peroxide in the presence of glucose oxidase; thereafter, hydrogen peroxide and *o*-dianisidine formed a colored product and finally, oxidic *o*-dianisidine reacted with sulfuric acid to form a more stable pink product, which was measured at 540 nm. The membrane-bound activities were expressed as mg glucose/g protein/min.

### Statistical analysis

Quantitative real-time PCR results were analyzed using a relative quantitative method (ΔΔC_t_) (Livak and Schmittgen 2001). Standard curves were obtained using a tenfold serial dilution of pooled total RNA. All the data were presented as the relative mRNA expression (mean ± SD). Data of membrane-bound trehalase activities and relative mRNA expression were evaluated for normality and homogeneity of variance. Trehalase activity and gene expression levels were analyzed using one-way ANOVA with Statistica 7.0 software. Multiple comparisons of the means were conducted using Tukey’s test. Differences between the means were deemed to be significant at *P* < 0.05.

## Results

### Structure of HaTreh1-5 and HaTreh2 proteins

These three Trehs were named *HaTreh1*-*5* (KX349223), *HaTreh2*-*like* (KX349224), and *HaTreh2* (KX349225). *HaTreh1*-*5*, *HaTreh2*-*like,* and *HaTreh2* cDNAs had open reading frames of 1761, 1662, and 1902 bp, respectively. *HaTreh1*-*5* cDNA encoded a protein of 586 amino acids with a predicted mass of approximately 69.47 kDa and a pI of 9.20 (Fig. S1A), whereas *HaTreh2*-*like* and *HaTreh2* encoded proteins of 553 and 633 amino acids with predicted mass of approximately 63.46 and 73.66 kDa, and pIs of 5.52 and 6.31, respectively (Fig. S1B, C).

**Fig. 1:**
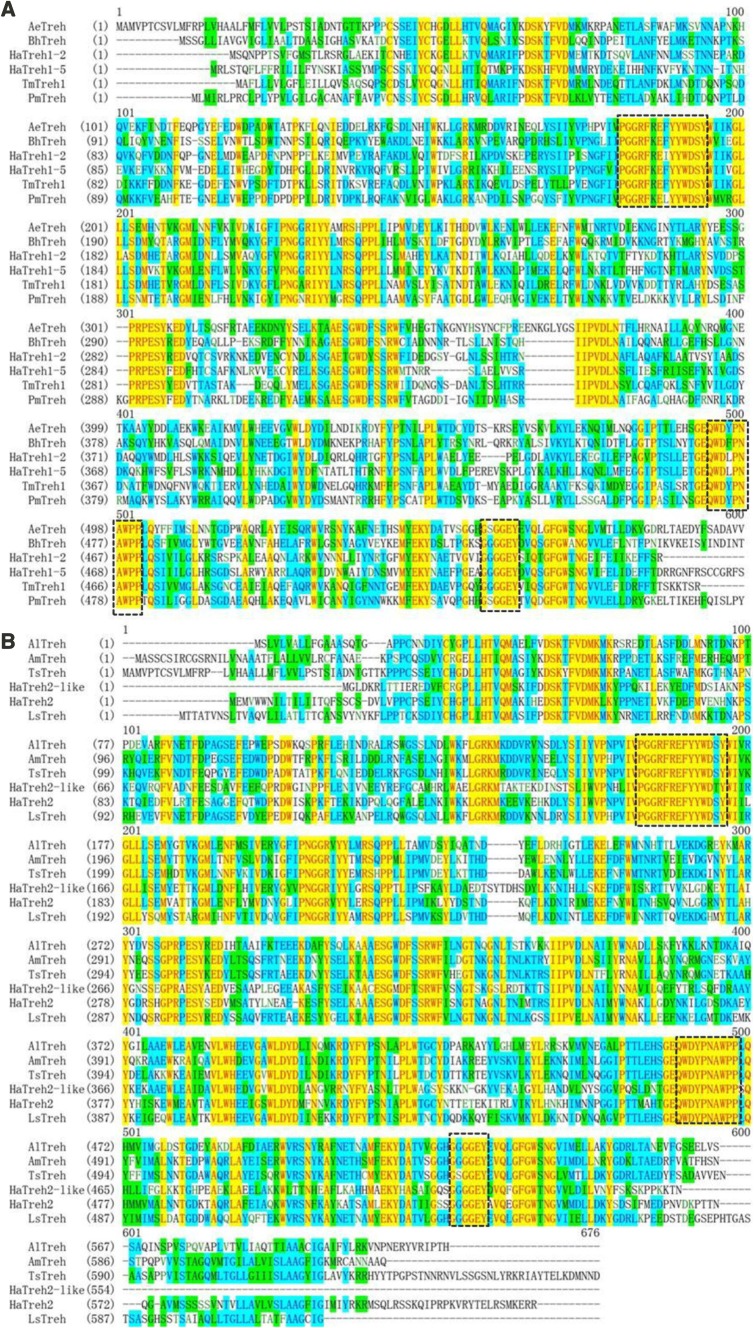
Alignment of Treh1 and Treh2 protein sequences from different insect species. **a** Alignment of HaTreh1-5 (KX349223), HaTreh1-2 (FJ501961), TmTreh1 (*T. molitor*, P32359), PmTreh1 (*Papilio machaon*, KPJ08342), AeTreh1 (*Acromyrmex echinatior*, EGI57245), and BhTreh1 (*Bombus hypocrite*, AHN15422) using Vector NTI 9.0 software. **b** Alignment of HaTreh2-like (KX349224), HaTreh2 (KX349225), AlTreh2 (*Apolygus lucorum*, AGL34007), LsTreh2 (*L. striatellus*, AFL03410), and TsTreh2 (*Trachymyrmex septentrionalis*, KYN38309) using Vector NTI 9.0 software. Highly conserved regions are shown in yellow and sky blue. Insect trehalase signatures and conserved sequences are boxed

The three new HaTreh proteins contained two specific motifs; HaTreh1-5 and HaTreh2 had the same specific motif “PGGRFKEFYYWDSY,” whereas HaTreh2-like contained “PGGRFREFYYEDTY.” Moreover, the HaTreh2-like and HaTreh2 had the same specific motif “QWDYPNAWPP,” whereas HaTreh1-5 had “QWDLPNAWPP.” Besides these specific motifs, the three new trehalase proteins contained the highly conserved glycine-rich (GGGGEY) region (Fig. 1) (Tang et al. 2012). Alignment of the proteins from a few other insect Trehs revealed that Treh1s had some conserved motifs. It was observed that all the insect Treh1s had one of the highly conserved motifs, “DSK*FVD”, “PGGRFK/RE*YYWDSYW”, or “IIPVDLN”, and also included “PRPESY” (Fig. 1a), Treh2s had “DSKT/YFVDMK”, “LGRKM”, “PNGGR”, and “WLDYD” motifs (Fig. 1b).

### Phylogenetic and multiple protein alignment analyses

**Fig. 2:**
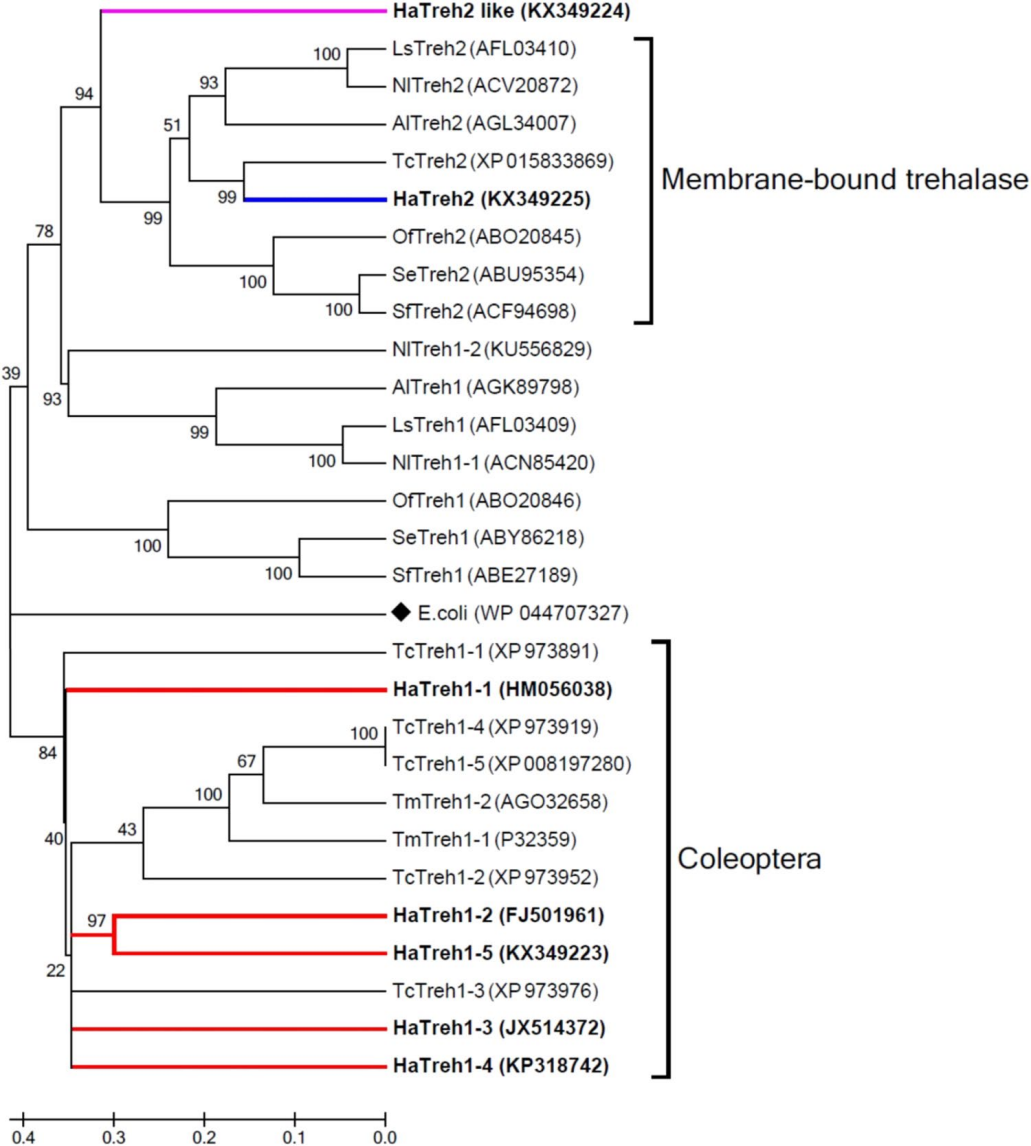
Phylogenetic tree constructed using the amino acid sequences of some known soluble and membrane-bound insect trehalases. Full-length amino acid sequences were aligned using the Mega 6.0 program. A bootstrap analysis was carried out and the robustness of each cluster was verified using 1000 replicates. Values at the cluster branches indicate the results of the bootstrap analysis. The soluble trehalase family proteins were LsTreh1 and LsTreh2 (*L. striatellus*), AlTreh1 and AlTreh2 (*Apolygus lucorum*), SeTreh1 and SeTreh2 (*S. exigua*), OfTreh1 and OfTreh2 (*O. fuscidentalis*), TcTreh1s and TcTreh2 (*T. castaneum*), NlTreh1s and NlTreh2 (*N. lugens*), SfTreh1 and SfTreh2 (*Spodoptera frugiperda*), TmTreh1s (*T. molitor*), and EcTreh (*E. coli*). 1-1, 1-2, 1-3, 1-4, and 1-5 show that the species has few of the different soluble trehalases. Characters in parentheses are the GenBank accession numbers

Some of the insect soluble and membrane-bound trehalase proteins were employed for phylogenetic analysis using MEGA6.0 software. The results showed that Treh1 and Treh2 proteins could be easily distinguished and that trehalases from the coleopteran insects could be assigned to the same subgroup (Fig. 2). *H. axyridis* and *T. castaneum* had five soluble trehalase proteins, and *T. molitor* had two soluble trehalases; twelve trehalases could be assigned to the same subgroup. The other soluble trehalases assigned to this subgroup, included trehalase of *Escherichia coli*, which formed a single member out-group. The membrane-bound trehalase proteins were assigned to another subgroup that excluded HaTreh2-like, which formed a single subgroup (Fig. 2). However, HaTreh2-like did not have the transmembrane domain near the C terminus and only have a transmembrane-like domain, but it was very similar to the other insect Treh proteins, especially HaTreh2 (Fig. 1b); therefore, it was named HaTreh2-like. We found a total of five trehalases in *H. axyridis*, namely *HaTreh1*-*1*, *HaTreh1*-*2*, *HaTreh1*-*3*, *HaTreh1*-*4*, and *HaTreh1*-*5*; all these belonged to the soluble trehalase cluster and were associated with *TmTreh* and *TcTreh1*.

*HaTreh1*-*5*, *HaTreh2*-*like*, and *HaTreh2*, showed 30.30–50.27, 31.57–51.37, and 32.77–65.66% identity to the other known *Treh* family genes. The soluble trehalase *Treh1* family could be clearly distinguished from the membrane-bound trehalase forms from different insects (Fig. 1). HaTreh2-like and HaTreh2 had the lowest identity with HaTreh1-3, which was 31.57 and 32.77%, respectively, and was even lower than the *E. coli* Treh protein (Table 2). HaTreh2-like had the highest identity of 51.37% with HaTreh2; it also had a higher identity with Treh2 than with Treh1 in the same insect. HaTreh1-5 was most identical to HaTreh1-like (50.27% identity) and TmTreh1-2 (50.50% identity), HaTreh2-like was most identical to HaTreh2 (51.37% identity) and TcTreh2 (50.82% identity), whereas HaTreh2 was most identical to TcTreh2 (65.66% identity) and LsTreh2 (58.63% identity) (Table 2).

**Table 2:** The related information of *Treh* gene family and the identity (%) to *HaTreh1*-*5*, *HaTreh2*-*like* and *HaTreh2*

Gene names	Species	Number of amino acids	Genebank numbers	Identity (%) to *HaTreh1-5*	Identity (%) to *HaTreh2-like*	Identity (%) to *HaTreh2*
HaTreh1-1	*Harmonia axyridis*	554	HM056038	44.71	41.79	44.33
HaTreh1-2	*H. axyridis*	553	FJ501961	50.27	41.04	42.91
HaTreh1-3	*H. axyridis*	547	JX514372	35.12	31.57	32.77
HaTreh1-4	*H. axyridis*	558	KP318742	44.93	41.48	40.69
HaTreh1-5	*H. axyridis*	586	KX349223	–	38.63	41.08
HaTreh2-like	*H. axyridis*	553	KX349224	38.63	–	51.37
HaTreh2	*H. axyridis*	633	KX349225	41.08	51.37	–
TcTreh1-1	*Tribolium castaneum*	541	XP973891	42.86	45.63	47.37
TcTreh1-2	*T. castaneum*	548	XP973952	45.86	42.67	45.47
TcTren1-3	*T. castaneum*	563	XP973976	44.78	42.86	43.76
TcTreh1-4	*T. castaneum*	553	XP973919	48.99	45.27	47.24
TcTreh1-5	*T. castaneum*	585	XP008197280	48.38	45.27	47.08
TcTreh2	*T. castaneum*	603	XP015833869	40.63	50.82	65.66
TmTren1-1	*Tenebrio molitor*	555	P32359	48.82	42.19	44.04
TmTreh1-2	*T. molitor*	558	AGO32658	50.00	42.70	46.45
NlTreh1-1	*Nilaparvata lugens*	546	ACN85420	40.30	43.39	43.58
NlTreh1-2	*N. lugens*	501	KU556829	37.85	38.04	40.64
NlTreh2	*N. lugens*	665	ACV20872	40.38	48.26	55.40
LsTreh1	*Laodelphax striatellus*	602	AFL03409	38.32	41.03	41.93
LsTreh2	*L. striatellus*	618	AFL03410	39.93	49.27	58.63
AlTreh1	*Apolygus lucorum*	643	AGK89798	39.54	43.75	50.49
AlTreh2	*A. lucorum*	617	AGL34007	42.35	48.91	54.73
OfTreh1	*Omphisa fuscidentalis*	581	ABO20846	40.00	41.47	43.78
OfTreh2	*O. fuscidentalis*	647	ABO20845	37.79	48.08	55.29
SeTreh1	*Spodoptera exigua*	585	ABY86218	36.63	39.08	41.83
SeTreh2	*S. exigua*	645	ABU95354	37.39	47.99	55.56
SfTreh1	*Spodoptera frugiperda*	587	ABE27189	36.68	39.82	42.56
SfTreh2	*S. frugiperda*	647	ACF94698	36.87	47.81	55.39
EcTreh	*Escherichia coli*	565	WP_044707327	30.30	32.35	33.15

### Developmental expression pattern of *HaTreh1*-*5* and *HaTreh2s*

**Fig. 3:**
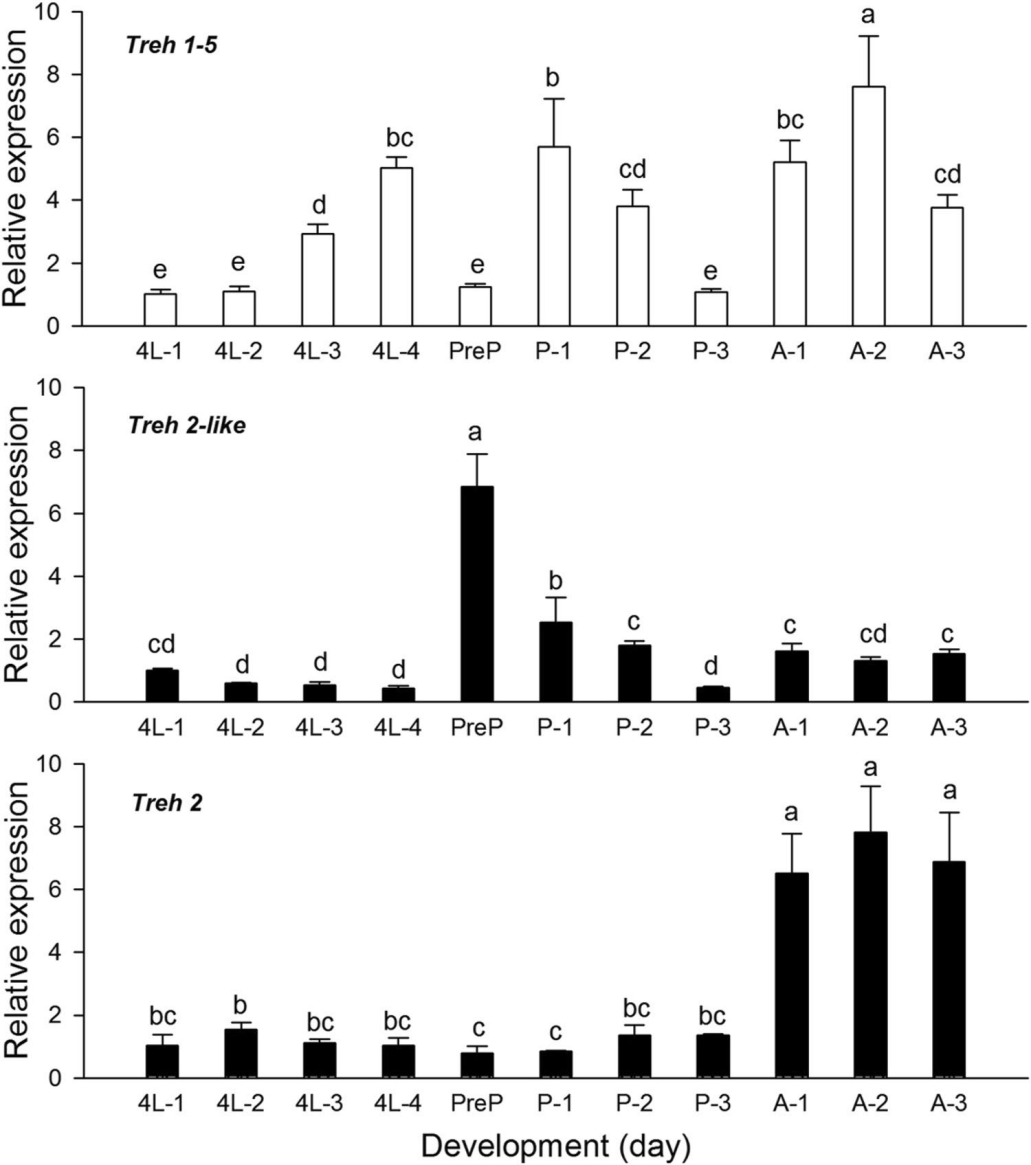
Expression levels of *HaTreh1*-*5*, *HaTreh2*-*like*, and *HaTreh2* mRNAs relative to rp49 expression as measured using qRT-PCR. Each point represents the mean ± SEM from three independent experiments. The developmental expression pattern of *HaTreh1*-*5*, *HaTreh2*-*like*, and *HaTreh2* cDNAs is represented. The indicated age of the insects is as follows: 4L1, the first day of the fourth larva; 4L2, the second day of the fourth larva; 4L3, the third day of the fourth larva; 4L4, the fourth day of the fourth larva; PreP, pre-pupa which was the first stage of pupa; P-1, the first day of pupa; P-2, the second day of pupa; P-3, the third day of pupa; A-1, the first day of adult; A-2, the second day of adult, and A-3 the third day of adult

The expression of *HaTreh1*-*5* was different during the different developmental stages. It increased from the first to the fourth day during the fourth larval stage, and decreased from the first to the third day in the pupal stages; the expression was higher during the previous 3 days of the adults (Fig. 3). The results showed that the expression of *HaTreh2*-*like* genes was at low level during the fourth larval stages and it reached the highest level at pre-pupae stage, and was followed by a decrease from the pre-pupal stage to the third day of pupa (Fig. 3). A uniform expression was observed during the 3 days of the adult stage. Although the expression pattern of *HaTreh2* was different from that of *HaTreh1*-*5* and *HaTreh2*-*like* genes, it had relatively lower expression levels from the first day of fourth instar larvae to the third day of pupal stage, and higher expression levels were observed during the adult stages (Fig. 3). Moreover, the expression of *HaTreh2*-*like* was higher in the pre-pupal stage whereas that of *HaTreh2* was higher in the adult stages (Fig. 3).

### Effects of cooling and re-warming process on the membrane-bound trehalase activity

**Fig. 4:**
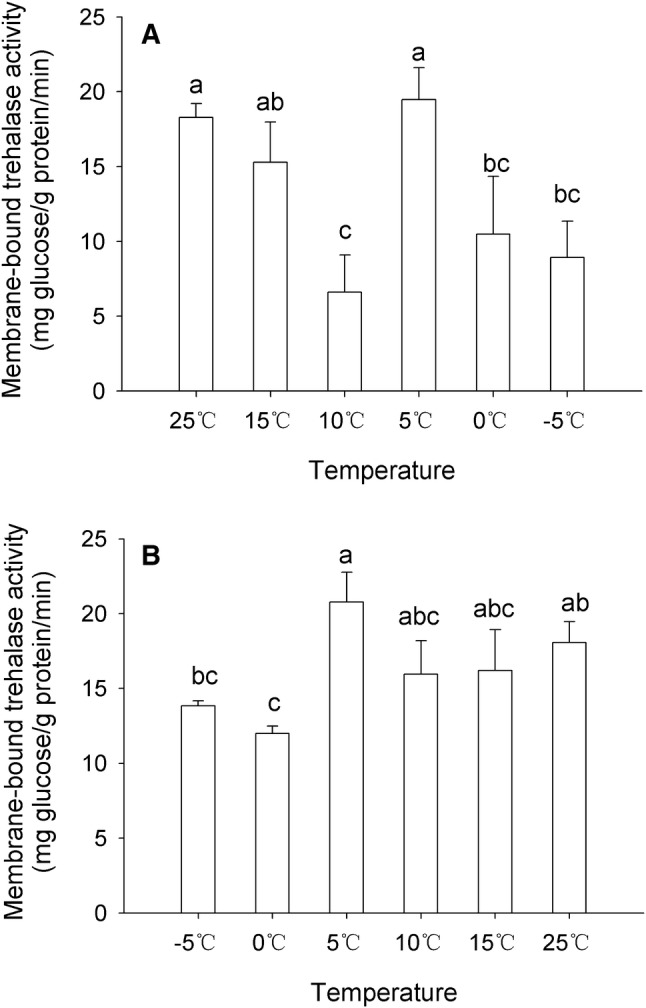
Trehalase activities in *H. axyridis* during cooling (**a**) and warming (**b**) conditions. Membrane-bound trehalase activities were examined in (**a**) an environment that was gradually cooled from 25 to − 5 °C and (**b**) an environment that was gradually warmed from − 5 to 25 °C. Bars with different letters indicate significant differences (*P* < 0.05). Data are presented as mean ± SD (*n* = 3)

Significant differences were observed in the activity of membrane-bound trehalases under the environments that were cooled or warmed. In the environment cooled from 25 to − 5 °C, the activity decreased in two steps, from 25 to 10 °C and from 5 to − 5 °C; the activity was higher at 25 and 5 °C than at 10, 0, and − 5 °C (Fig. 4a). The Treh2 activity was increased from 10 to 5 °C, it showed that more trehalose was needed for degradation to glucose for providing energy. In contrast, in the environment warmed from − 5 to 25 °C, the enzyme activity increased compared to the activity at − 5 and 0 °C; the highest activity was observed at 5 °C (Fig. 4b). From 5 to 25 °C, the trehalase activity showed a relatively higher value compared to that at − 5 and 0 °C (Fig. 4b). Both, under the cooling and warming environments, the membrane-bound trehalase activity reached a maximum at 5 °C.

### Changes in the expression of three *Treh* mRNAs during rapid cold and re-warming treatment

**Fig. 5:**
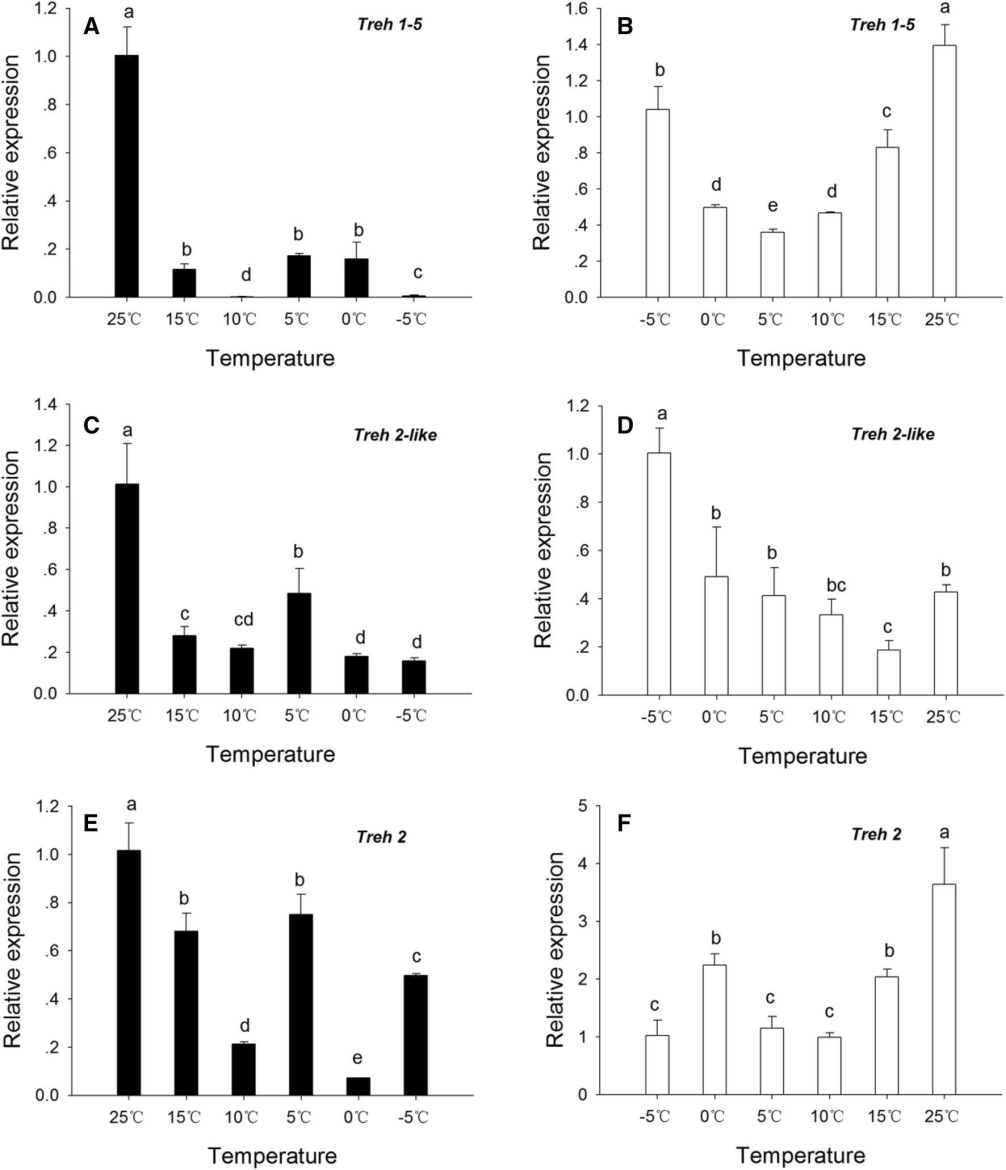
Relative expression of *HaTreh1*-*5* (**a**), *HaTreh2*-*like* (**c**), and *HaTreh2* (**e**) identified from *H. axyridis* determined by real-time PCR in an environment gradually cooled from 25 to – 5 °C. The relative expression of *HaTreh 1*-*5* (**b**), *HaTreh2*-*like* (**d**), and *HaTreh2* (**f**) identified from *H. axyridis* were tested by real-time PCR in an environment gradually warmed from − 5 to 25 °C. All the mRNA levels were measured relative to the *rp49* mRNA levels. Data are presented as mean ± SD (*n* = 3)

In the cooling treatment groups, the mRNA expression of *HaTreh1*-*5*, *HaTreh2*-*like*, and *HaTreh2* decreased significantly from 25 to − 5 °C, the expression of all the three genes being highest at 25 °C (Fig. 5a, c, e). The expression of all the three genes was also higher at 5 °C compared to the expression at other treatment temperatures. The expression of *HaTreh1*-*5* was very low at 10 and − 5 °C (Fig. 5a), whereas that of *HaTreh2* was low at 10 and 0 °C (Fig. 5e); the expression of *HaTreh2*-*like* was low at all the temperatures except at 25 and 5 °C (Fig. 5c). The expression patterns of the three genes were different in the warming treatment groups. *HaTreh1*-*5* and *HaTreh2*-*like* expression had a similar trend; it decreased first followed by an increase. The expression of *HaTreh1*-*5* was significantly lower at 5 °C than at other temperatures (Fig. 5b). The expression of *HaTreh2*-*like* was lowest at 15 °C (Fig. 5d). The expression of HaTreh2 increased from − 5 to 25 °C; it was high at 0 and 15 °C and reached the highest level at 25 °C (Fig. 5f).

## Discussion

Insect trehalase proteins have the same or similar characteristic motifs (“PGGRFREFYYWDSY”, “QWDYPNAWPP” and “GGGGEY”), which are essential for catalytic activity and substrate-binding functions (Mitsumasu et al. 2005; Shukla et al. 2015; Tang et al. 2008, 2012; Nardelli et al. 2019). All these features suggest that it is a relatively conserved protein family (Forcella et al. 2010). HaTreh1-3 showed low identity with HaTreh1-5 (35.12%), HaTreh2-like (31.57%), and HaTreh2 (32.77%) (Table 2), which could also be the reason for HaTreh1-3 being present on a separate branch in the Treh1 phylogenetic tree compared to the other Treh1s and for HaTreh1-3 showing only 28–40% identity to other known *Treh1* family genes (Tang et al. 2014). HaTreh1-1, HaTreh1-3, and HaTreh1-4 were present on a single branch in the phylogenetic tree (Fig. 2). They also possessed some other conserved motifs, including “PVD/ELN” and “PRPESY” (Fig. 1), similar to the other HaTreh1s (Tang et al. 2014). Moreover, most of the insects only have one Treh1 and one Treh2 proteins, with only the coleopteran insects *T. castaneum* and *H. axyridis* having more than three soluble trehalase genes (Shi et al. 2016; Tang et al. 2014, 2016). In *L. migratoria*, it also has four trehalase genes, and a *Treh2*-*like* gene has been found, which is expressed in all tissues, such as integument, midgut, Malpighian tubules, fat body and so on. In addition, the expression levels of *Treh1* and *Treh2* in tissues are specific (Liu et al. 2016). In the same time, a *Treh2*-*like* gene was also found in *H. axyridis* in our study, and this is the first-found *Treh2*-*like* gene in insect. We believe that trehalases are very important proteins and the *Treh* genes evolved through time; *Treh2*-*like* might represent the gene for a protein, intermediate in form, between the soluble and membrane-bound trehalases. Therefore, *Treh2*-*like* could be a new class of trehalase genes, then more and more similar *Treh2*-*like* genes will be found.

The pI of soluble trehalases in insects is known to be around 4.5 and that of the membrane-bound forms is around 6.5 (Tang et al. 2012). However, pIs more than 6.5 have also been reported for some insect trehalases. It was reported that the pI of HaTreh1-3 was 8.88 and was similar to that of TcTreh1-2 (8.81), TcTreh1-3 (7.02), and DpTreh1-3 (6.56) (Shi et al. 2016; Tang et al. 2014). The pI of HaTreh1-5 has also been reported to be 9.20 and hence is positively charged, favoring adsorption onto the negatively charged matrix, also being higher than that of HaTreh1-4 (6.32), HaTreh2-like (5.52), and HaTreh2 (6.31). Almost all the insect trehalases have one signal peptide or putative cleavage sites, ranging from 1 to 30 aa in length (Tang et al. 2012), an exception being AmTreh2 (NM_001112671) with a 35 aa signal peptide (Mori et al. 2009), while two Treh1 and one Treh2 in *L. migratoria* did not have the signal peptide structure (Liu et al. 2016). The signal peptide in the coleopterans contains about 20 aa, HaTreh1-1 to HaTreh1-4 have a 20- or 21-aa-long signal peptide, and HaTreh1-5 and HaTreh2 have signal peptides with 24 and 20 aa, respectively (Fig. S1A, C). It is interesting to note that HaTreh2-like did not have a signal peptide (Fig. S1B), this result is similar to *L. migratoria Treh1* and *Treh2* genes (Liu et al. 2016).

Many soluble insect trehalases were observed to be expressed in the midgut, fat body, epidermis, ovary, and Malpighian tubules of larvae in *T. molitor* (Gomez et al. 2013; Takiguchi et al. 1992), *B. mori* (Kamei et al. 2011; Kamimura et al. 1999), *Pimpla hypochondriaca* (Parkinson et al. 2003), as well as four soluble and one membrane-bound trehalases expressed in cuticle, fat body, midgut and Malpighian tubules of the last larvae in *T. castaneum* (Tang et al. 2016). The expression of *Treh1* and *Treh2* in *S. exigua* was mainly observed in the last instar larvae, pupae, and adults (Chen et al. 2010). Most of the insects need two kinds of trehalases to use trehalose during the different developmental stages, but they only have one soluble and membrane-bound trehalase gene. However, *H. axyridis* has more than seven trehalase genes. Therefore, various trehalase genes play their specific functions at different stages of life. It can be found from the expression of five *HaTreh1*, one *HaTreh2*-*like* and one *HaTreh2* during the development stages (Shi et al. 2016; Tang et al. 2014). *HaTreh1*-*1* and *HaTreh1*-*2* might play the role in the larval stage whereas *HaTreh1*-*3* might have a role in stages from larva to adult (Tang et al. 2014). Moreover, *HaTreh1*-*3*, *HaTreh1*-*5*, and *HaTreh2* are known to have roles in the adult stage (Fig. 3) (Shi et al. 2016). *HaTreh2*-*like* may also have a role during the pre-pupal stages (Fig. 3). In short, *HaTreh1*-*5*, *HaTreh2*-*like*, and *HaTreh2* have unique expression patterns through the developmental stages. These genes, including the other four *HaTreh1* genes, co-regulate the changes in the trehalose content and energy production to adapt to the development processes in *H. axyridis*.

Previous studies on the protective function of trehalose under different stress conditions, including desiccation, dehydration, heating, freezing, and oxidation, have been reported (Bale and Hayward 2010; Crowe et al. 1984; Elbein et al. 2003; Khani et al. 2007; Shimada 1984). In insects, trehalose participates in both the homeostasis and development as does the blood sugar (Silva et al. 2004; Thompson 2003); it is involved in processes such as flight metabolism, cold tolerance, and so on (Tatun et al. 2008a, b). In *H. axyridis*, the trehalose contents significantly increased when adults were placed at 5, 0, and − 5 °C during the cooling process (Shi et al. 2016), suggesting that it can protect *H. axyridis* from the cold stress, it was induced and as a cryoprotectant at 5 °C condition. A previous study showed that soluble trehalase activity could be gradually suppressed with the decrease in temperature (Shi et al. 2016). In the present study, we detected the membrane-bound trehalase activity (Treh2) during the cooling and warming processes. We observed that 5 °C was the turning point of the change of Treh2 activity, and the enzyme activity was the strongest at 5 °C (Fig. 4). Shi et al. found that trehalose content was the highest level at 5 °C during cooling process, it showed some sugars, such as glycogen, has transformed into trehalose (Shi et al. 2016). These results also indicate that a temperature of 5 °C may be critical for the adaptation of insects to cold; at this temperature, the insect not only requires more energy from trehalose, it also needs more trehalose for the cold-resistance property of this sugar. For this reason, trehalases play an important role in insects transforming energy stored in different sugars. These results suggest that Treh2 activity plays a key role in the adaptation to the cooling process.

Trehalose-6-phosphate synthase gene expression has also been reported to increase from 25 to 5 °C during the cooling process (Qin et al. 2012). During the process of warming, the Treh1 activity showed an initial increase, followed by a decrease, and was increased again at 25 °C (Shi et al. 2016). Treh2 activity was increased and was maintained at high levels throughout (Fig. 4b) in conjunction with a decrease in the trehalose content, signifying that Treh2 might be playing a role in the warming process. However, the expression level of *TPS*, trehalose content and Treh activities increased when trehalase inhibitor of validamycin injected into *N. lugens* (Tang et al. 2017).

Trehalose is an energy source for insects providing energy for the movement and development of insects. It is rapidly used when the insect faces stress conditions (Tang et al. 2014; Thompson 2003). In contrast to cold acclimation during overwintering, which is induced over several weeks or months, rapid cold hardening of arthropods can be induced by a short exposure (several minutes or hours) to low temperatures (from 0 to 5 °C) or through gradual cooling (from 0.1 to 1 °C min^−1^) over a range of temperature (Lee et al. 1987; Lee 1991). The changes in the trehalase activity can be achieved through the modulation of the expressions of trehalase genes. For example, our results indicate that the relative expressions of *Treh2*-*like* and *Treh 2* genes were consistent with changes in Treh2 activity during cooling (first decline, then increase at 5 °C, then decrease). However, during the warming process, the trends between them were different, and we speculated that it may be related to other physiological processes not found in *H. axyridis*. In the present study, we observed changes in the expression of *HaTreh1*-*5* and *HaTreh2* mRNAs in the adults of *H. axyridis* subjected to rise and drop in temperature over a short period of time (Fig. 5). The expression of *HaTreh1*-*4* has also been reported to decrease under cooling process (Shi et al. 2016) and the Treh1 activity was also observed to decrease. These results suggest that *HaTreh1*-*4* and *HaTreh1*-*5* play the main role during this process. *HaTreh2*-*like* and *HaTreh2* could be sharing a similar function during the cooling process as revealed by their expression pattern and the Treh2 activity (Figs. 4a, 5c, e). The expression of all the three *HaTreh* mRNAs showed a decreasing trend during the cooling process, indicating that all the Trehs play a protective role during low temperatures although the expression of those *Treh* genes are suppressed (Fig. 5). The expression of *TPS* mRNA has previously been shown to increase initially and then decease following the drop in temperature in *H. axyridis* adults (Qin et al. 2012). Moreover, higher *TPS* expression can increase the content of trehalose only if the activities of trehalase remain unchanged or decreased because of the decrease in *Treh* mRNA levels. It is also reported that the trehalose content increased when single *TPS* gene was knocked down (Tang et al. 2010; Yang et al. 2017). *H. axyridis* is an important predatory ladybug and a natural enemy of agricultural and forestry pests. It is very necessary to apply it to biological control, but low-temperature storage is a difficult problem. The results of Figs. 4 and 5 provide a theoretical basis for screening storage temperatures and the ability to improve resistance to low temperatures through cold training, such as 5 °C as a potential storage temperature. In addition, it seems that the rapid low-temperature stress of *H. axyridis* is more conducive to the accumulation of trehalose than the gradual cooling, because Treh2 activity has been maintained at a high level.

Trehalase is well known to be important in insect growth, development, and molting processes, and is involved in providing energy (Tang et al. 2008; Matsuda et al. 2015; Shukla et al. 2015; Yoshida et al. 2016). Moreover, the roles of different *Treh1* genes are not same, although the function of trehalases is to degrade trehalose to glucose (Tang et al. 2008; Chen et al. 2014; Shukla et al. 2015). The reason for the presence of more than five soluble trehalases, and a Treh2-like trehalase in some insects and understanding of their specific functions in development and growth, especially for tissue-specific distribution and its function on disaccharide–monosaccharide transition during different tissues at different time and conditions, would need further investigation.

## Conclusion

Our study provides a theoretical basis for revealing the different physiological roles of three novel trehalase genes in the growth, cooling and re-warming process of *H. axyridis*. Results indicate that *H. axyridis* adults can accumulate trehalose early in the process of adaptation to rapid cold hardening. The physiological mechanisms underlying rapid cold hardening may also be associated with the accumulation of a cryoprotectant, such as glycerol and other low molecular weight polyhydric alcohols and sugars (Lee et al. 1987; Watanabe 2002; Zhao et al. 2008). The functional studies related to physiological adaption of insects having different *HaTreh* genes need to be undertaken in future. It should involve a combined approach in which the expression of *Treh* genes during the cooling or heating process is inhibited by RNA interference (RNAi) and the function of individual *Treh* genes is also knocked down by dsRNAs injection or by other methods.

## Electronic supplementary material

Below is the link to the electronic supplementary material.

Supplementary material 1 (DOCX 933 kb)
