# Supplementary material for: Three novel trehalase genes from Harmonia axyridis (Coleoptera: Coccinellidae): cloning and regulation in response to rapid cold and re-warming
Source: 3 Biotech. 2019 Aug 6;9(9):321. doi: 10.1007/s13205-019-1839-9 (PMC6684730; doi:10.1007/s13205-019-1839-9)
Supplement: Supplementary file 1 — Supplementary material 1 (DOCX 933 kb) [file 13205_2019_1839_MOESM1_ESM.docx]

**
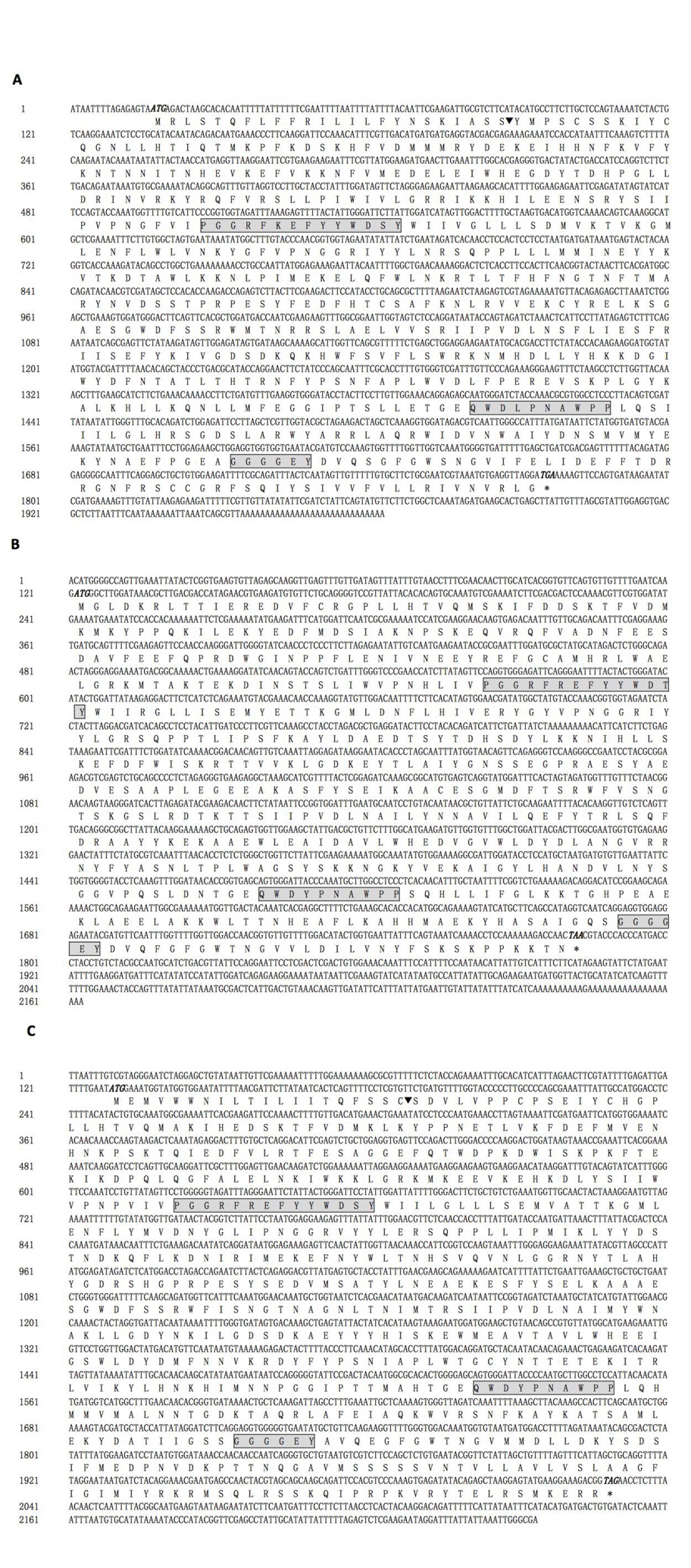
**

**Figure S1 Analysis of nucleotide and deduced amino acid sequences of *HaTreh1-5*, *HaTreh2-like*, and *HaTreh2*.** Initiation and termination codons are indicated in bold and italicized font; the termination codon before the first Met is also indicated in bold and italics. The amino acid residues between the arrowheads (1-24 or 1-20) represent the signal peptide and putative cleavage site for HaTreh1-5 and HaTreh2, respectively. Three trehalase signatures or conserved sequences (amino acid residues PGGRFK/REFYYWDS/TY, QWDL/YPNAWPP, and GGGGEY) are boxed and shaded in grey. The nucleotide sequences reported in this paper have been submitted to GenBank (accession numbers: KX349223, KX349224, and KX349225). A: HaTreh1-5; B: HaTreh2-like; **C:** HaTreh2.
